# A Galacto-Oligosaccharides Preparation Derived From Lactulose Protects Against Colorectal Cancer Development in an Animal Model

**DOI:** 10.3389/fmicb.2018.02004

**Published:** 2018-08-31

**Authors:** Javier Fernández, F. J. Moreno, Agustín Olano, Alfonso Clemente, Claudio J. Villar, Felipe Lombó

**Affiliations:** ^1^Research Unit “Biotechnology in Nutraceuticals and Bioactive Compounds-BIONUC”, Departamento de Biología Funcional, Área de Microbiología, Universidad de Oviedo, Oviedo, Spain; ^2^Instituto Universitario de Oncología del Principado de Asturias, Oviedo, Spain; ^3^Instituto de Investigación Sanitaria del Principado de Asturias, Oviedo, Spain; ^4^Instituto de Investigación en Ciencias de la Alimentación (CIAL-CSIC), Madrid, Spain; ^5^Estación Experimental del Zaidín (EEZ-CSIC), Granada, Spain

**Keywords:** prebiotic, colorectal cancer, prevention, galacto-oligosaccharides, gut microbiota

## Abstract

Colorectal cancer (CRC) is one of the most common neoplasias worldwide, and its incidence is increasing. Consumption of prebiotics is a useful strategy in order to prevent this important disease. These nutraceutical compounds might exert protective biological functions as antitumors. In order to test the chemopreventive effect of GOS-Lu (galacto-oligosaccharides derived from lactulose) prebiotic preparation against this cancer, an animal model (*Rattus norvegicus* F344) was used. In this model, two doses of azoxymethane (10 mg/kg) and two treatments with dextran sodium sulfate (DSS) were administered to the animals. Animals were fed for 20 weeks, and either control drinking water or drinking water containing 10% (w/w) GOS-Lu prebiotic preparation was provided to them. Animals were sacrificed after those 20 weeks, and their digestive tract tissues were analyzed. The results revealed a statistically significant reduction in the number of colon tumors in the GOS-Lu cohort with respect to control animals. Metagenomics sequencing was used for studying colon microbiota populations, revealing significant reductions in populations of pro-inflammatory bacteria families and species, and significant increases in interesting beneficial populations, such as *Bifidobacterium*. Therefore, oral administration of the prebiotic GOS-Lu preparation may be an effective strategy for preventing CRC.

## Introduction

Prebiotics have been recently redefined as a substrate that is selectively utilized by host microorganisms conferring a health benefit (Gibson et al., 2017). Prebiotics are typically metabolized by bifidobacteria and lactobacilli and their major beneficial effects seem to occur in the large intestine due to the slow transit of the substrates susceptible of fermentation and their effects on microbial diversity and metabolic fingerprinting, which play an important role in host health (Bindels et al., 2015). These effects include growth inhibition of potential pathogens, immune response stimulation, modulation of intestinal epithelial cells and production of short-chain fatty acids (SCFAs) as metabolic endpoints of carbohydrate fermentation. The most abundant SCFAs are acetate, butyrate and propionate (which constitute more than 95% of total SCFA content), playing a key role in maintaining intestinal homeostasis. These metabolites are linked to specific health aspects at gut level and elsewhere in the body, including pathogen exclusion, colonocyte function, epithelial cell proliferation and differentiation, energy intake and control of body weight, levels of secondary bile acids, mineral absorption (Ca, Fe, and Mg), cholesterol biosynthesis, glucose metabolism and insulin sensitivity (Canfora et al., 2015; Fernández et al., 2015; Koh et al., 2016).

Prebiotic compounds are non-digestible oligosaccharides with various origins and chemical characteristics. They differ in the chain length, monosaccharide composition, linkage type and degree of branching (Clemente, 2014). The most deeply studied oligosaccharides are human milk oligosaccharides (HMO), inulin, fructo-oligosaccharides (FOS), the disaccharide lactulose and galacto-oligosaccharides (GOS) derived from lactose. Recently, novel galacto-oligosaccharides derived from lactulose (GOS-Lu) have demonstrated their potential as prebiotic compounds. These can be enzymatically produced by transgalactosylation of lactulose (4-*O*-β-D-galactopyranosyl-D-fructose) using β-galactosidases from different microbial sources (Martínez-Villaluenga et al., 2008; Cardelle-Cobas et al., 2012; Díez-Municio et al., 2014). Scientific evidences supporting their potential application as emerging prebiotic ingredients exerting beneficial effects on the gastrointestinal tract have been gathered (Moreno et al., 2014). Thus, GOS-Lu have shown to be selective for bifidobacteria and lactic-acid bacteria following several *in vitro* fermentation studies (Cardelle-Cobas et al., 2008, 2009, 2011). This bifidogenic effect was further corroborated in growing rats fed 1% (w/w) of GOS-Lu (Hernández-Hernández et al., 2012), together with a significant and selective increase of *Bifidobacterium animalis* found in the caecum and colon sections (Marín-Manzano et al., 2013). *In vitro* (Ferreira-Lazarte et al., 2017) and *in vivo* (Hernández-Hernández et al., 2012) studies have revealed that GOS-Lu are significantly less digestible in rats than conventional GOS (enzymatically synthetized from lactose). The higher resistance to gastrointestinal digestion together with the presence of non-transgalactosylated lactulose, which is itself a prebiotic, instead of lactose suggests that GOS-Lu have a lower calorific content than conventional GOS products (Rastall, 2013). Another prebiotic-mediated beneficial effect ascribed to GOS-Lu is their capacity to improve iron absorption in an iron-deficient rat model (Laparra et al., 2014). Lastly, GOS-Lu has been reported to inhibit *in vitro* production of pro-inflammatory factors, such as TNF-α and IL-1β, by intestinal epithelial cells (Caco-2) stimulated by the pathogen *Listeria monocytogenes* CECT 935 (Laparra et al., 2013); in addition, GOS-Lu has been reported to exert preventive intestinal anti-inflammatory effects in the trinitrobenzenesulfonic acid model of rat colitis (Algieri et al., 2014).

Colorectal cancer (CRC) is one of the leading causes of cancer-related mortality worldwide in both men (after lung and prostate cancers) and women (after breast cancer), and it is expected to increase by 60% to more than 1.1 million deaths by 2030 (Merrill and Anderson, 2011; Bray et al., 2013; Ferlay et al., 2015; Arnold et al., 2017). CRC is a complex and heterogeneous disease that reflects a combination of hereditary (such as mutations in specific genes such as *apc*), environmental (such as tobacco, alcohol, etc.) and dietary factors (such as saturated fat, nitrosamines, benzopyrenes, low consumption of fruit and vegetables) (Jemal et al., 2011; Brenner et al., 2014). Accumulating data suggest that the gut microbiota, and particularly their metabolic end products, might exert a protective role against CRC development by influencing inflammation, DNA damage and apoptosis (Louis et al., 2014). Prebiotics have been showed to improve biomarkers associated to CRC, as they stimulate the growth and activity of gut beneficial bacterial populations (Gibson and Roberfroid, 1995; Roberfroid, 2007; Pompei et al., 2008; Bosscher et al., 2009), which generate diverse short-chain fatty acids (SCFAs) as acetate, propionate, butyrate, isobutyrate and valerate when feeding on these prebiotic fibers (Rumessen et al., 1990). Some of these SCFAs exert interesting antitumor properties, as they inhibit histone deacetylases, causing changes in the expression of diverse cell cycle key modulators, and inducing apoptosis in tumor colon cells (Roller et al., 2004a; Pool-Zobel, 2005; Verghese et al., 2005; Kim and Milner, 2007; Scharlau et al., 2009; Stein et al., 2012; Fernández et al., 2015).

Consequently, the aim of this work was to evaluate the potential chemopreventive effects of orally ingested GOS-Lu against CRC in an animal model (*Rattus norvegicus* F344). Tumors were chemically induced with azoxymethane (AOM) and dextran sodium sulfate (DSS). Diverse biochemical, physical and microbiological parameters were analyzed in these rats: body weight, number of hyperplastic Peyer’s patches, caecum weight, number of colon polyps and total tumor-affected area. The intestinal microbiota was also examined in the two animal cohorts (control rat feed, GOS-Lu), revealing significant differences.

## Materials and Methods

### Production and Characterization of GOS-Lu

Galacto-oligosaccharides derived from lactulose (Lu) were synthesized using a commercial lactulose preparation (670 g of lactulose per liter; Duphalac, Abbott Biologicals BV, Olst, Netherlands) and β-galactosidase from *Aspergillus oryzae* (16 U/mL; Sigma, St. Louis, MO, United States) (López-Sanz et al., 2015). The enzymatic reaction took place at pH 5.4, achieved after the addition of 3 mL of KOH 2M at 800 mL of Duphalac, and 50°C in an orbital shaker at 300 rpm for 24 h. Afterwards, the enzymatic reaction was stopped by heating at 110°C for 10 min. The resulting mixture contained 66% (w:w) of total carbohydrates.

The carbohydrate fraction was qualitatively and quantitatively determined by gas chromatography-flame ionization detector (GC-FID) as trimethyl silylated oxime (TMSO) derivatives following previous approaches (Cardelle-Cobas et al., 2009; Hernández-Hernández et al., 2012). The carbohydrate composition of GOS-Lu, whose main involved glycosidic linkage was β(1→6), was as follows: fructose (19.5%), galactose (12.4%), glucose (1.2%), lactulose (24.7%), GOS-Lu disaccharides (13.6%), GOS-Lu trisaccharides (22.6%), GOS-Lu tetrasaccharides (5.1%) and GOS-Lu pentasaccharides (1.0%).

### Animals and Experimental Design

Twenty male Fischer 344 rats (5 weeks old) were kept at the authorized facility No. ES330440003591 (University of Oviedo), and experiments were started after approval by the Ethics Committee of the Principado de Asturias (PROAE 16/2015).

Rats were separated in two groups of 10 animals each one. Groups 1 and 2 were fed *ad libitum* with universal feed (2014 Teklad Global 14% Protein Rodent Maintenance Harlan diet feed, Harlan Laboratories, Barcelona, Spain). The composition of this diet is the following one^1^: protein 14.3%, fat 4%, carbohydrate 48%, crude fiber 4.1%, neutral detergent fiber 18%, ash 4.7%, energy 2.9 kcal/g.

Group 1 received drinking water. Group 2 received 10% (w/w) GOS-Lu dissolved in the drinking water (average daily intake of GOS-Lu per rat was 2 g).

Rats number 9 and 10 of each group were kept free of CRC induction, as absolute control animals.

### Colorectal Cancer Induction and Monitoring

One week after the animals arrived, drinking water or 10% (w/w) GOS-Lu in drinking water was provided continuously. After 1 week of drinking the corresponding liquid, CRC was induced in eight rats from each cohort. The two other rats were kept free of CRC induction as absolute control animals. Azoxymethane (AOM, Sigma-Aldrich, Madrid, Spain) was used for CRC induction in 8 rats of each group. AOM was dissolved in sterile saline (0.9% w:v NaCl) at 2 mg/mL and injected intraperitoneally (10 mg/kg body weight). The AOM treatment was repeated 1 week after first injection (weeks 2 and 3). The 2 control rats per group received sterile saline injections.

Rats received 3% and 2% (w:v) dextran sodium sulfate (DSS, 40.000 g/mol, VWR) in drinking water during 7 days, on weeks 4 and 15, respectively. This ulcerative colitis challenge was repeated twice in order to reinforce the pro-carcinogenic exerted by AOM. All rats were sacrificed at weeks 20 (pneumothorax). During those 20 weeks, rats were monitored for stool consistency, rectal bleeding and body weight.

### Body Weight

Weight was measured along the 20 experimental weeks: arrival of animals (week 1), both AOM administrations (weeks 2 and 3), both DSS challenges (weeks 4 and 15), at week 6 and at sacrifice.

### Blood and Tissue Samples

At week 20, rats were anesthetized with isoflurane and sacrificed (pneumothorax). All caecums were weighed and frozen at -20°C.

Colon was opened along main axis, washed with PBS (phosphate buffer saline) and kept in 4% v:v formaldehyde (4°C). The tumors number (from 1 to 9.5 mm diameter) were counted in the colon mucosa. Tumor morphologies were annotated as pedunculated, circular, spherical, and plane irregular, in order to get the total tumor-affected area.

### GC-MS Quantification of SCFAs in Feces Using Deuterated Standards

Four hundred milligrams of frozen caecum feces were thawed and resuspended in 1,716 μL milli-Q H_2_O in 5 mL glass vials, homogenized in vortexed. Then, deuterated SCFAs standards were added as internal controls: deuterated acetate, butyrate, propionate and valerate (Cambridge Isotope Laboratories, United States), to a final concentration of 0.4 mM each one. Finally, 400 μL of 50% H_2_SO_4_ and 800 mg NaCl were added as well. This mixture was resuspended and 1 mL of ethyl acetate was added as extraction solvent. Samples were stirred for 1 h at 300 rpm and 25°C, and centrifuged for 5 min at 3500 rpm. 500 μL supernatants were taken to a new vial. This extraction was repeated twice.

The gas chromatography-mass spectrometry (GC-MS) equipment was an Agilent 7890A (Agilent Technologies), equipped with an inert crosslink mass selective detector (XL MSD) with triple-Axis detector. Acquisition was done using Chemstation software. The capillary chromatographic column was DB-FFAP (30 m, 0.25 mm ID, 0.25 μm film thickness). Helium was used as the carrier gas at 1 mL/min. Injection was made in splitless mode with an injection volume of 1 μL and an injector temperature of 200°C. A glass liner with a glass wool plug at the lower end of the liner was used to avoid the contamination of the GC column with non-volatile fecal material. A blank sample was inserted between experimental samples, to check for memory effects.

The column temperature was initially 50°C (1 min), then was increased to 150°C at 5°C/min, and finally to 230°C at 15°C/min (total time 20 min). The temperature of the ion source, quadrupole, and interface were 230, 150, and 220°C, respectively. Scanning ions were 45 and 76 *m/z* for deuterated propionic acid, 45 and 74 *m/z* for propionic acid, 43 and 73 *m/z* for isobutiric acid, 63 and 77 *m/z* for deuterated butyric acid, 60 and 73 *m/z* for butyric acid, 60 and 87 *m/z* for isovaleric acid, 63 and 77 *m/z* for deuterated isovaleric acid, 60 and 73 *m/z* for valeric acid and 60, 73, and 87 *m/z* for hexanoic acid. Identification of the different SCFAs was based on the retention time of standards and with the assistance of the Wiley 7 library.

### Genomic DNA Extraction and 16S Ribosomal RNA Sequencing for Metagenomics

E.Z.N.A.^®^ DNA Stool Kit (Ref. D4015-02, VWR, Madrid, Spain) was used for genomic DNA (gDNA) extraction (200 mg of frozen caecum feces). A BioPhotometer^®^ (Eppendorf, Madrid, Spain) was used for gDNA quantification, a prior step before preparing working solutions diluted to 6 ng/μL, which were needed for PCR amplification using the Ion 16^TM^ Metagenomics kit (Thermo Fischer Scientific, Madrid, Spain).

PCR amplicons were used to generate a library (Ion Plus Fragment Library kit for AB Library Builder^TM^ System, Cat. No. 4477597, Thermo Fischer Scientific, Madrid, Spain). The indexing of each sample was carried out with the Ion Xpress^TM^ Barcode Adapters 1-96 kit (Cat. No. 4474517, Thermo Fischer Scientific, Madrid, Spain). The ION OneTouch^TM^ 2 System and the ION PGM^TM^ Hi-Q^TM^ OT2 kit (Cat. No. A27739, Thermo Fischer Scientific, Madrid, Spain) was used for preparing the templates. The ION^TM^ PGM Hi-Q^TM^ Sequencing kit (Cat. No. A25592, Thermo Fischer Scientific, Madrid, Spain) on the ION PGM^TM^ System was used for metagenomics sequencing. The ION 318^TM^ v2 Chip (Cat. No. 4484355, Thermo Fischer Scientific, Madrid, Spain) was used.

### Phylogenetic Analysis

For each rat metagenomics, the consensus spreadsheet (ION Reporter software 5.6, Life Technologies Holdings Pte. Ltd., Singapore) included the percentages for each phylum, class, order, family or genus/species. These data were used in order to compare frequencies between experimental groups. Taxonomic adscription up to species level was performed using the QIIME 2 (v.2017.6.0) open-source bioinformatics pipeline. Analysis of the microbiome community was carried out using R software (v3.2.4): non-supervised multivariate analysis (PCA). For LDA analysis, tab-delimited files were generated in R and computed at family level using Galaxy. Graphical representation of Galaxy output included only discriminative features with logarithmic LDA score higher than 3. The reference library used was the Curated MicroSEQ(R) 16S Reference Library v2013.1; Curated Greengenes v13.5. The number of mapped reads (after the ignored ones due to less than 10 copies) per sample was always over 60.000. Total number of reads was always over 110.000. Counts were normalized by sum scaling. All raw metagenomics data have been deposited at NCBI SRA database (submission ID SRP155959).

### Statistical Methods

Shapiro–Wilk’s test was used for calculating the Gaussian distribution of the different variables. Data were then expressed as the mean value ± SEM (standard error of mean). *t*-Test and other parametric methods were used for showing these data. Levene’s test was used for checking the similarity of variances. In the case of normal distribution, unpaired *t*-Test (when variances were similar) or Welch *t*-Test (when variances were not similar) were used for determining the statistical differences. In the case of no normal distribution, the non-parametric Mann–Whitney test was used for determining the statistical differences among cohorts.

GraphPad Prism software version 7 (GraphPad Software, San Diego, CA, United States) was used for the graphical representations: a *p*-value < 0.05 was considered statistically significant.

## Results

### Effect of GOS-Lu on Body Weight

**FIGURE 1:**
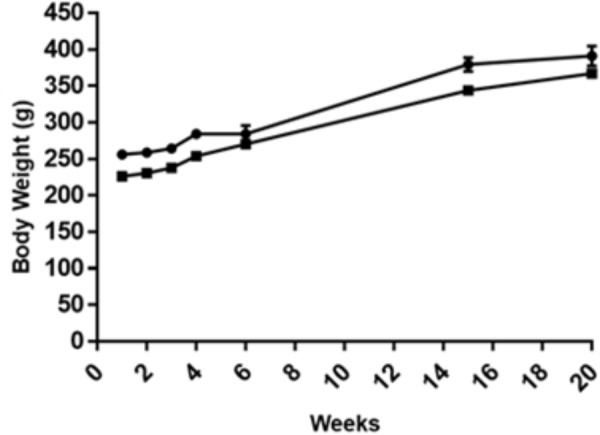
Body weight along the experimental time for the eight animals with CRC induction in the two groups: control (circles), GOS-Lu (squares). Body weight was measured at weeks 1, 2, 3, 4, 7, 15, and 20.

Body weight gain were similar for all rat groups along the 20 experimental weeks (the first AOM challenge for CRC induction took place at week 2) (see **Supplementary Table S2**). When the animals were sacrificed, the mean value for the control cohort was 391.1 ± 40.5 g whereas for the GOS-Lu cohort was 367.1 ± 17.3 g (**Figure 1**).

The second DSS challenge caused the death of rat number 3 (control cohort) due to intense rectal bleeding. This transitional ulcerative colitis process was a pro-inflammatory step necessary to increase the final tumor numbers and sizes.

### Effect of GOS-Lu on Caecum Weight

**FIGURE 2:**
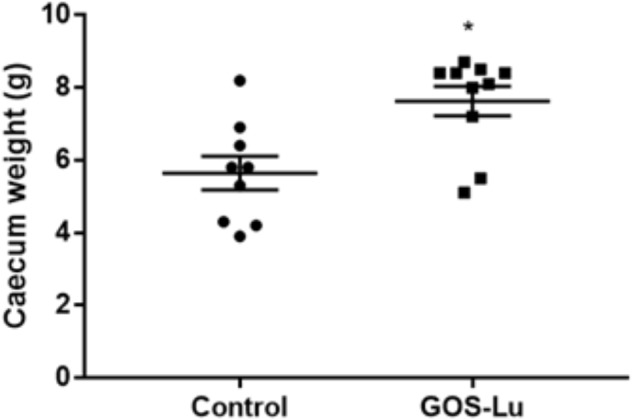
Mean of caecum weight in grams for each cohort.

Statistically significant differences in the caecum weight values were observed between the control cohort and the GOS-Lu cohort. These mean values were increased in the GOS-Lu cohort (7.63 ± 0.4 g) with respect to the control cohort (5.64 ± 0.4 g) and these differences were statistically significant (*p*-values 0.001) (**Figure 2**) (see **Supplementary Table S2**). Caecums from GOS-Lu cohort showed a 35.28% increase, due to the stimulation of bacterial populations caused by the presence of prebiotic compounds. In rodents, fermentation of prebiotic compounds starts in this organ.

### Effect of GOS-Lu on Number of Polyps and Tumor-Affected Area

**FIGURE 3:**
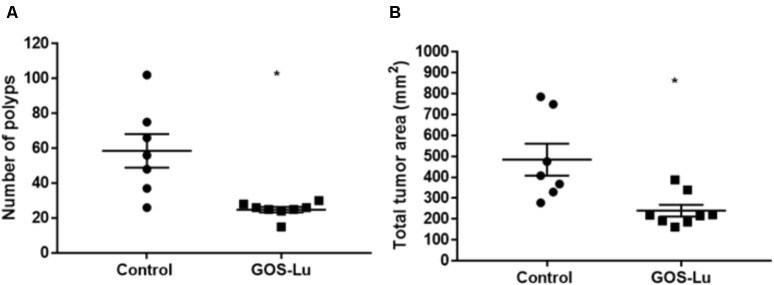
Measurements of colon polyps. **(A)** Average number of colon polyps. **(B)** Average sum of polyp areas.

The colonic mucosa from each animal was analyzed for the number of polyps. Polyp diameter ranged from 1 to 9.5 mm (see **Supplementary Table S2**). A statistically significant difference was observed between rats in the control cohort and those in the GOS-Lu cohort. Polyps number decreased in the case of the GOS-Lu group (24.8 ± 1.5) with respect to the control group (58.5 ± 9.5) and this difference was statistically significant (*p*-value 0.0022). The GOS-Lu cohort showed a drastic 57.5% reduction in the number of polyps (**Figure 3A**).

Each polyp area was also calculated depending on its shape, computing the total tumor area for each rat. A statistically significant reduction was observed in the mean affected area of the GOS-Lu cohort (240 ± 28.1 mm^2^) with respect to the control cohort (484.8 ± 76.6 mm^2^). Therefore, the GOS-Lu cohort showed a 50.4% reduction in this parameter with respect to the control cohort (**Figure 3B**).

### Effect of GOS-Lu on SCFAs Production

**FIGURE 4:**
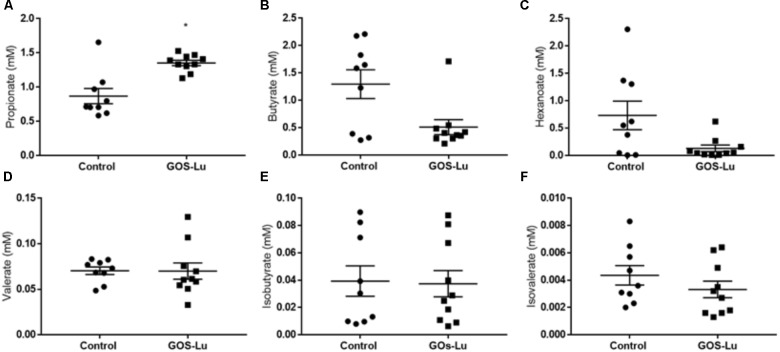
Cecal short chain fatty acids concentrations. **(A)** Propionate. **(B)** Butyrate. **(C)** Hexanoate. **(D)** Valerate. **(E)** Isobutyrate. **(F)** Isovalerate.

Cecal production of SCFAs is important, as these compounds show interesting antitumor properties regarding CRC prevention. Six SCFAs were identified and quantified in cecal content: propionate, butyrate, isobutyrate, valerate, isovalerate, and hexanoate. Propionate concentrations in control (0.86 ± 0.1) and GOS-Lu (1.35 ± 0.03) cohorts showed a statistically significant difference, with a 56.9% higher production in the GOS-Lu cohort (*p*-value 0.003) (**Figure 4A**). Concentrations of butyrate in control (1.29 ± 0.2) and GOS-Lu (0.51 ± 0.1) cohorts, of hexanoate in control (0.73 ± 0.2) and GOS-Lu (0.13 ± 0.06) cohorts, of valerate in control (0.07 ± 0.004) and GOS-Lu (0.06 ± 0.008) cohorts, of isobutyrate in control (0.04 ± 0.01) and GOS-Lu (0.03 ± 0.009) cohorts, and of isovalerate in control (0.004 ± 0.0007) and GOS-Lu (0.003 ± 0.0006) cohorts showed no statistically significant differences (**Figure 4**) (see **Supplementary Table S2**).

### Effect of GOS-Lu on Intestinal Microbiota

Average phyla compositions of the two cohorts showed important differences between both animal groups (**Table 1**). The main differences between these cohorts is a 39.18% higher proportion in *Bacteroidetes* in the GOS-Lu cohort, a 19.35% reduction in *Firmicutes* populations in GOS-Lu cohort, and the near absence of *Proteobacteria* in the rats of the control cohort (0.34%), whereas *Proteobacteria* is the third most common phylum in the GOS-Lu cohort (9.49%) (**Table 1**). The predominant phylum in both cohorts was *Firmicutes* (69.14% in the control cohort, 55.76% in the GOS-Lu cohort) (**Table 1**). GOS-Lu increased the abundance of *Actinobacteria* and decreased *Tenericutes* (**Table 1**) (see **Supplementary Table S1**).

**Table 1:** Average percentage composition of intestinal microbiota at phylum level for the two cohorts studied.

Percentage of:	Control	GOS-Lu	*p*-value
*Actinobacteria^∗^*	1	2.58	0.008
*Bacteroidetes^∗^*	22.51	31.33	0.035
*Firmicutes^∗^*	69.14	55.76	0.042
*Proteobacteria*	0.34	9.49	–
*Synergistetes^∗^*	0.06	0.12	0.029
*Tenericutes^∗^*	0.31	0	0.002
*Verrucomicrobia*	0.53	0.68	–
*Deferribacteres*	0.07	0.03	–
*Spirochaetes*	0.01	0	–


*The ^∗^ indicates statistical significant differences*.

**FIGURE 5:**
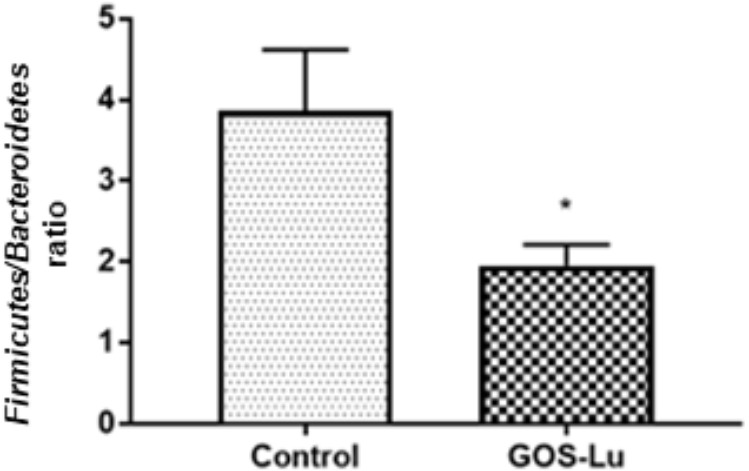
Graphical representation of the *Firmicutes/Bacteroidetes* ratio in both rat cohorts. ^∗^ Means a statistical significant difference between both cohorts.

The *Firmicutes/Bacteroidetes* ratio, described as an obesity microbiome marker, showed that it was significantly higher in the control cohort (3.86 ± 0.75) than in GOS-Lu cohort (1.95 ± 0.25) (*p*-value 0.03) (**Figure 5**).

**FIGURE 6:**
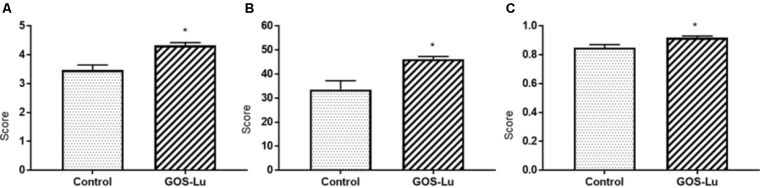
Graphical representation of the diversity indexes associated to the gut microbiota. **(A)** Shannon. **(B)** Chao. **(C)** Simpson.

As a general description of the gut microbiota composition, the Shannon diversity index was increased in the GOS-Lu cohort. This index is 3.47 (±0.17) for control cohort and 4.32 (±0.09) for GOS-Lu cohort (statistical significant differences, with a *p*-value of 0.0004) (**Figure 6A**). The Chao and the Simpson diversity indexes indicate an increase in the gut microbiota diversity in GOS-Lu cohort as well. Chao index is 33.56 (±3.66) for control cohort and 46.2 (±1.13) for GOS-Lu cohort (statistical significant differences, with a *p*-value of 0.0086) (**Figure 6B**). Simpson index is 0.8494 (±0.02) for control cohort and 0.9177 (±0.01) for GOS-Lu cohort (statistical significant differences, with a *p*-value of 0.0086) (**Figure 6C**). A homogeneous distribution between all rats in each cohort was found at phyla level.

**FIGURE 7:**
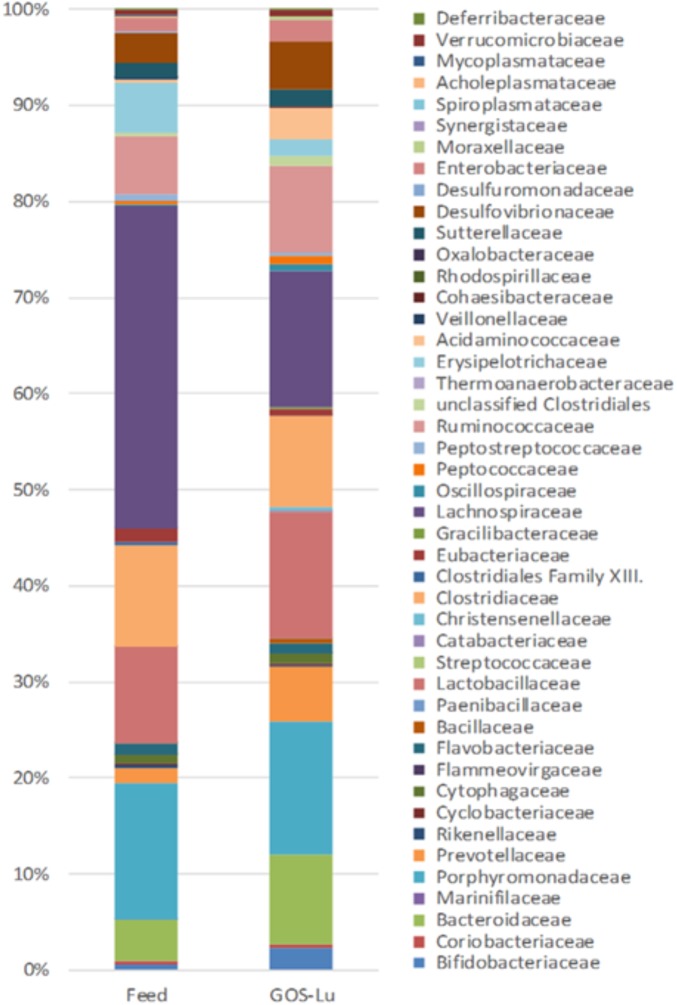
Differences in average intestinal microbiota composition at the family level for both cohorts.

**FIGURE 8:**
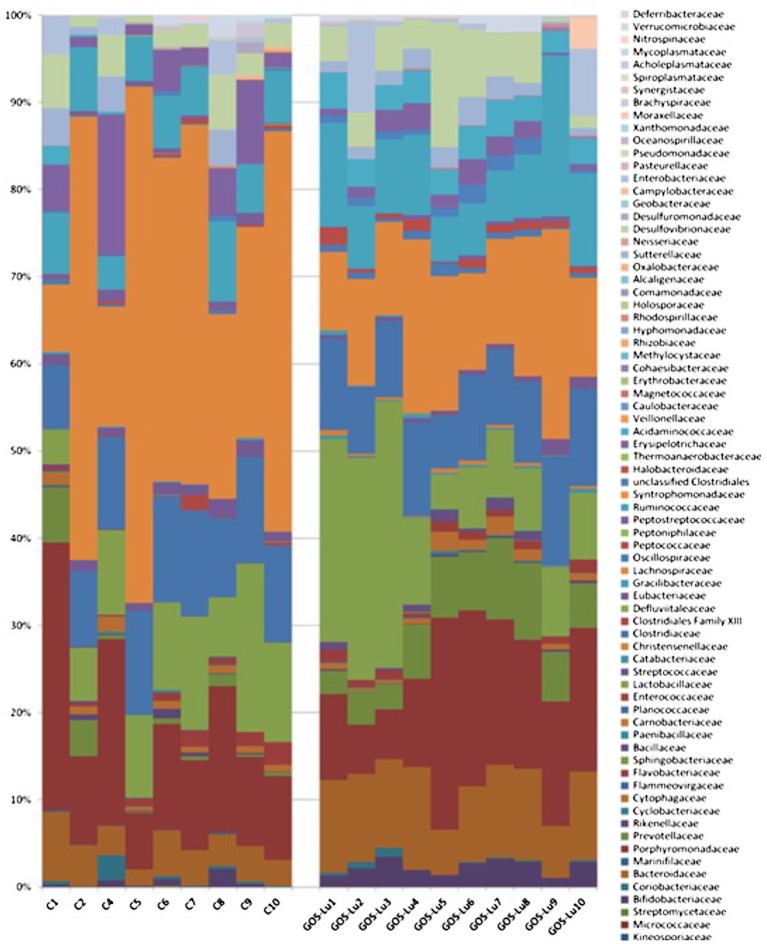
Intestinal microbiota composition at the family level in the rats belonging to the control cohort (C1–C10), and in the rats belonging to the GOS-Lu cohort (GOS-Lu1 to GOS-Lu10).

At the family level, in the control cohort, the most abundant families were *Lachnospiraceae* (33.47%), *Porphyromonadaceae* (14.17%), *Clostridiaceae* (10.56%), *Lactobacillaceae* (9.96%), and *Bacteroidaceae* (4.22%) (**Figure 7**). In the case of GOS-Lu cohort, the most abundant ones were *Lachnospiraceae* (14.18%), *Porphyromonadaceae* (13.78%), *Lactobacillaceae* (13.06%) *Clostridiaceae* (9.49%), and *Bacteroidaceae* (9.40%) (**Figure 7**). These compositions at family level were homogeneous between animals in each cohort (**Figure 8**) (see **Supplementary Table S1**).

**FIGURE 9:**
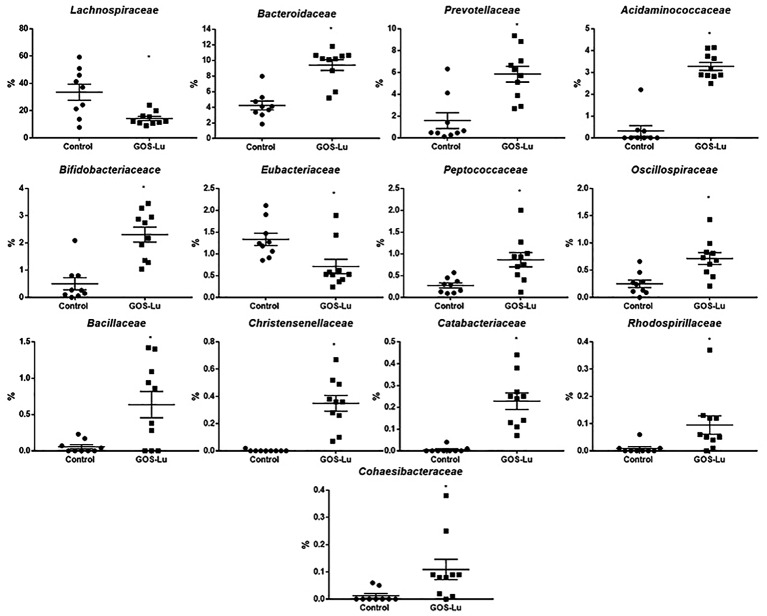
Families showing statistical significant differences between the rats belonging to the control and GOS-Lu cohorts.

Also at family level, statistically significant differences were found mainly in the case of *Lachnospiraceae* (33.47% ± 5.87 in control cohort and 14.18% ± 1.49 in GOS-Lu cohort), *Bacteroidaceae* (4.22% ± 0.57 in control cohort and 9.40% ± 0.68 in GOS-Lu cohort), *Prevotellaceae* (1.58% ± 0.72 and 5.84 ± 0.002, respectively), *Acidaminococcaceae* (0.32% ± 0.24 and 3.27% ± 0.18, respectively), *Bifidobacteriaceae* (0.50% ± 0.22 and 2.30% ± 0.27, respectively), *Eubacteriaceae* (1.33% ±0.14 and 0.71% ± 0.16, respectively), *Peptococcaceae* (0.27% ± 0.05 and 0.86% ± 0.16, respectively), *Oscillospiraceae* (0.25% ± 0.06 and 0.71% ± 0.10, respectively) and *Acholeplasmataceae* (0.23% ± 0.16 and 0%, respectively) (**Figure 9**).

The observed statistical significant increase in *Actinobacteria* phylum (**Table 1**) was mainly due to an increment in the *Bifidobacteriaceae* family (0.5% in control cohort, 2.31% in GOS-Lu cohort) (**Figures 7, 9**). In the case of *Tenericutes* phylum, its observed decrease was mainly due to a reduction in *Acholeplasmataceae* family (0.23% in control cohort, 0% in GOS-Lu cohort) (**Figure 7**).

The *Bacteroidaceae* (4.22% in control cohort, 9.4% in GOS-Lu cohort) and *Prevotellaceae* (1.59% in control cohort, 5.84% in GOS-Lu cohort) families were the remarkable groups from the *Bacteroidetes* phylum that showed an increase in the GOS-Lu cohort, compared to the control group (**Figures 7, 9**).

The decrease in *Firmicutes* phylum which took place in the GOS-Lu cohort was mainly due to a reduction of the *Lachnospiraceae* family (33.47% in control cohort, 14.18% in GOS-Lu cohort) and to a lesser extent, to a reduction in *Eubacteriaceae* family (1.33% in control cohort, 0.71% in GOS-Lu cohort) as well (**Figures 7, 9**).

**FIGURE 10:**
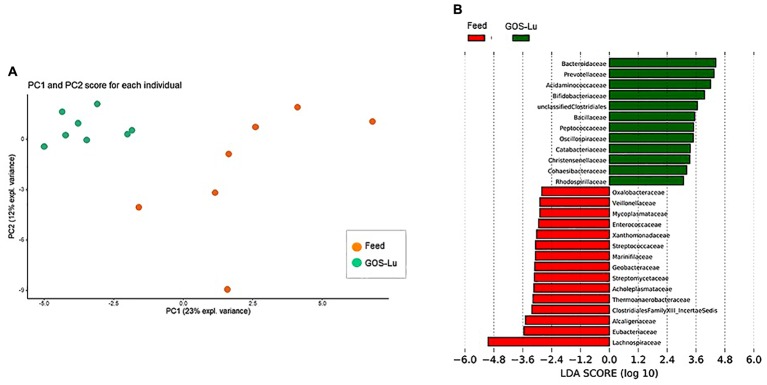
**(A)** Gut microbiota PCA cluster analysis. **(B)** LDA analysis showing the families that better discriminate between both cohort conditions.

Despite the decline of *Firmicutes* phylum in the GOS-Lu cohort, several families showed a small but significant increase in their numbers, such as *Bacillaceae, Oscillospiraceae, Peptococcaceae, Catabacteriaceae, Christensenellaceae*, unclassified *Clostridiales* and *Acidaminococcaceae* (**Figures 7, 9**). PCA of gut microbiota composition divided the animals in two clusters, indicating differences in the gut microbiota composition associated to both dietary interventions, feed and GOS-Lu supplementation (**Figure 10A**). Bacterial genera and species with significant differences in their relative abundances between the GOS-Lu and the control groups are indicated in **Table 2** and the LDA analysis is represented in **Figure 10B**: in total, 27 families explain in a significant way the two types of diet. Finally, the relative abundance at genus and species levels was studied. Most significant differences were associated to higher proportion of some genera in the GOS-Lu cohort, such us *Bifidobacterium, Bacteroides, Parabacteroides* and others (**Table 2**). Only a few significant genera and species showed a reduction in GOS-Lu cohort, such as *Lactobacillus intestinalis, Turicibacter* spp., and *Desulfovibrio* spp. (**Table 2**).

**Table 2:** Statistical significant differences in intestinal microbiota composition at the genus and species level for the rats belonging to the control and GOS-Lu cohorts.

Genus	Species	Control (mean value)	GOS-Lu (mean value)	*p*-value
*Bifidobacterium*		0.50	2.31	0,0002
	*animalis*	0.01	0.46	0,0003
*Bacteroides*		0.53	6.98	<0,0001
	*eggerthii*	0.03	2.72	<0,0001
	*massiliensis*	0.03	0.43	0,0001
	*thetaiotaomicron*	0.01	0.22	0,0008
	*uniformis*	0.16	1.40	0,001
	*vulgatus*	0.04	1.04	<0,0001
	*xylanisolvens*	0.00	0.27	0,0004
*Parabacteroides*		0.30	1.39	<0,0001
	*distasonis*	0.22	0.46	0,0134
	*merdae*	0.05	0.87	<0,0001
*Paraprevotella*		0.03	4.20	<0,0001
	*clara*	0.00	0.13	0,0007
*Lactobacillus*	*intestinalis*	0.24	0.06	0,041
*Anaerostipes*		0.01	0.40	<0,0001
*Blautia*		1.84	6.19	0,0071
	*producta*	0.60	1.13	0,0052
	*wexlerae*	0.00	0.69	0,0325
*Dorea*		0.01	0.61	0,0018
*Lachnoclostridium*	*clostridioforme*	0.04	0.80	0,0002
*Faecalibacterium*		0.73	1.88	0,0009
	*prausnitzii*	0.13	0.35	0,0028
*Ruminococcus*		0.27	1.26	0,0023
	*gauvreauii*	0.12	0.38	0,0296
*Subdoligranulum*		0.03	0.13	0,0024
*Flavonifractor*		0.06	0.95	<0,0001
	*plautii*	0.05	0.95	<0,0001
*Eubacterium*	*dolichum*	0.00	0.16	<0,0001
*Turicibacter*		0.52	0.00	<0,0001
*Phascolar-ctobacterium*		0.32	3.28	<0,0001
	*succinatutens*	0.25	2.70	<0,0001
*Bilophila*		1.66	4.35	0,0422
	*wadsworthia*	1.54	3.99	0,034
*Desulfovibrio*		1.42	0.53	0,0178
	*C21_c20*	0.18	0.00	0,0031


## Discussion

In this work, the antitumor effect of GOS-Lu as a prebiotic compound was studied in an animal model where CRC was generated using AOM/DSS. This GOS-Lu prebiotic preparation contains some amounts of monosaccharides (33.1%) and lactulose (24.7%) from the enzymatic synthesis reaction. It contains also prebiotic disaccharides (13.6%), trisaccharides (22.6%), tetrasaccharides (5.1%), and pentasaccharides (1%). In total, these prebiotics (galactosyl-fructoses) account for 42.3%, a nearly two-fold proportion to lactulose. GOS-Lu was used here without any prior purification step (including monosaccharides and the precursor lactulose substrate) in a similar way to the conventional GOS used by food industry. This is a common practice for prebiotics acting as food ingredients (for instance, following their addition to infant formulae) to make their use more cost-effective and feasible, since the removal of mono- and disaccharides would make the manufacturing process substantially more expensive. Therefore, GOS-Lu was tested without any previous purification to provide a more straightforward potential industrial application.

Rat number 3 in the control group died during the second DSS challenge, as this transient ulcerative colitis stage causes rectal bleeding. No significant differences on the body weight gain was observed between both animal cohorts during the 20 experimental weeks.

At week 20th, all 19 surviving animals were sacrificed and diverse histological measures were analyzed: caecum weight, number of colon tumors, and total tumor area in the colon mucosa (**Figures 2, 3**).

Caecum weight was significantly increased in animals from GOS-Lu cohort, an indication of the prebiotic properties of this preparation, as caecum is the main section of the digestive tract where prebiotic fermentative process occurs, being carried out by gut microbiota. Monosaccharides present in GOS-Lu preparation are absorbable carbohydrates at the small intestinal level, and they do not reach significant concentrations at colon level. Therefore, these free sugars (glucose and fructose) should not have any major impact in the outcome of these CRC animal model results. Regarding lactulose in the GOS-Lu preparation, it is well established that it undergoes fermentation rapidly in the proximal colon (Venema et al., 2003; Undseth et al., 2015). In consequence, the additional interest of GOS-Lu relies on the fact that it possesses lower fermentation rates as compared with lactulose because longer carbohydrate chains are normally fermented slower (Perrin et al., 2002), so their action takes place in the distal parts of the colon more efficiently (Díez-Municio et al., 2014). In this context, it is worth to mention a previous work demonstrating that GOS-Lu showed a better anti-inflammatory profile than lactulose in the trinitrobenzenesulfonic acid rat animal model of colitis, derived from the improvement of the luminal microbiota balance and a greater SCFA production (Algieri et al., 2014).

In the presence of prebiotics, some bacterial populations flourish in the caecum, contributing to a larger organ (**Figure 2**) (Roller et al., 2004b; Wang et al., 2010; Chen et al., 2011). GOS-Lu has been already described as prebiotic, and in this work, its daily intake of 2 g per rat has been able to clearly increase caecum weight (Moreno et al., 2014).

A factor which is directly correlated with the presence of prebiotics in the caecum is the bacterial fermentation of these compounds in this organ, giving rise to diverse SCFAs. In this study, a 56.9% increase in the caecum production of propionate was clearly observed, in a statistically significant way (**Figure 4A**). For the other detected SCFAs, these differences were not observed (**Figure 4**). These results will be commented below, after describing the changes in intestinal microbiota composition between both cohorts.

The number of colon tumors and the total tumor area in the colon mucosa clearly demonstrated a protective effect of GOS-Lu supplementation with respect to CRC. There was a statistically significant tumors number and area reduction (57.5 and 50.4, respectively) in the GOS-Lu cohort (**Figure 3**).

After analyzing these histological parameters, the intestinal microbiota composition was determined through metagenomics 16S ribosomal RNA (rRNA) sequencing of cecal content. The taxonomic diversity of a given metagenomics sample can be calculated using diverse alpha-diversity indexes. In this work, Shannon, Chao and Simpson indexes show that caecum bacterial richness in the animals belonging to GOS-Lu cohort is higher than in control cohort animals (**Figure 6**).

Analysis of gut microbiota at phylum level showed important differences between both cohorts (**Table 1**). More specifically, a high *Firmicutes/Bacteroidetes* ratio has been proposed as a microbiome marker for obesity and type II diabetes (two inflammatory and metabolic conditions) in human and animal studies (Ley et al., 2005, 2006). In this study, animals from GOS-Lu cohort showed increased *Bacteroidetes* and reduced *Firmicutes* populations, lowering significantly this ratio, a protective effect according to literature.

At the family level, the significant increase observed in the GOS-Lu cohort in *Bacteroidetes* phylum was mainly due to an increase in the abundance of *Bacteroidaceae* family (more specifically *Bacteroides* genus) and *Prevotellaceae* family (*Paraprevotella* genus), together with an increase in the *Parabacteroides* genus (**Table 2** and **Figures 7, 9**). These three genera have been described as good propionate producers (Polansky et al., 2016). *Phascolarctobacterium* genus (which belongs to the *Acidaminococcaceae* family, a *Firmicutes*) is also a good propionate producer, and it is increased in GOS-Lu cohort as well. These three *Bacteroidetes* genera and one *Firmicutes* genus are the main responsible ones of the high increase in propionate production in the GOS-Lu cohort (**Table 2** and **Figures 9, 10A**), which is a plausible explanation for the strong reduction in tumor numbers in this prebiotic cohort (**Figure 3**), as it has been described before for this SCFA (Ooi et al., 2010).

The significant reduction in *Firmicutes* phylum is due to lower population levels in members of the *Lachnospiraceae* family in general (**Figures 7, 9**). However, some genera of this family are slightly more abundant in the GOS-Lu cohort (*Lachnoclostridium, Anaerostipes, Dorea, Blautia*). This *Lachnospiraceae* family has been involved in high butyrate production (Polansky et al., 2016), and its reduction might be the main responsible for the reduced butyrate levels observed in the GOS-Lu cohort. One of the species showing an important reduction within *Lachnospiraceae* family is the mucolytic bacteria *Ruminococcus gnavus*, which has been reported to be more abundant in Crohn’s Disease (CD) and Intestinal Bowel Disease (IBD) patients (Png et al., 2010; Willing et al., 2010; Joossens et al., 2011) and may play an important role in inducing chronic intestinal inflammation (Eun et al., 2014). *Eubacteriaceae* family (from *Firmicutes* phylum) is also reduced in GOS-Lu cohort. This can have also a protective role against pro-inflammatory conditions, as an increase in this family has been described in dogs suffering IBD (Omori et al., 2017).

Three *Firmicutes* families are more abundant in the GOS-Lu cohort. These are *Oscillospiraceae, Christensenellaceae*, and *Ruminococcaceae* (*Faecalibacterium*, and *Subdoligranulum* genera) (**Table 2**). This is remarkable, as *Faecalibacterium* has been described as an anti-inflammatory genus, as it secretes metabolites able to block NF-kB activation, an important pro-inflammatory cellular mediator which is also involved in colon carcinogenesis (Sokol et al., 2006; Han et al., 2016; Huang et al., 2017). In a similar way, *Christensenellaceae* family has been associated with beneficial effects in the host, as lower obesity risk (Goodrich et al., 2014).

At the level of the *Actinobacteria* phylum, GOS-Lu cohort shows an increase in this bacteria, mainly due to more abundant populations of *Bifidobacteriaceae* family, and specially of *Bifidobacterium* genus, being associated to health benefits including protection against pro-inflammatory gut conditions but also against CRC (**Table 1** and **Figure 9**). This protection against CRC has been associated to NF-kB repression by these probiotic bacteria (Roller et al., 2004a; Kim et al., 2010; Celiberto et al., 2017; Paveljšek et al., 2018).

Finally, regarding *Proteobacteria* phylum, *Desulfovibrio* genus (*Desulfovibrionaceae* family) populations are reduced in GOS-Lu cohort (**Table 2**). This bacteria have been associated to apoptosis induction in the colon mucosa in *in vitro* models and to a pro-inflammatory changes in UC patients, such as decreased sulphation of mucin, as a result of the metabolic activity of these bacteria (Lennon et al., 2014; Coutinho et al., 2017). Also, this bacterial genus has been associated to increasing levels of damage (also toward CRC) at the mucosal level, caused by reduction of the mucin barrier, the natural protection against luminal microbiota pro-inflammatory challenges (Song et al., 2018). However, another member of this family, *Bilophila wadsworthia*, is increased in GOS-Lu cohort. Although this species has been associated to a pro-inflammatory effect, some reports associate this mainly to the presence of low levels of the anti-inflammatory IL-10 (such as those found in IL-10-/- mice mutants colon mucosa) (Rennick et al., 1997; Devkota et al., 2012; Devkota and Chang, 2015). Therefore, in a wild type rat, as in this work, the pro-inflammatory effect of *B. wadsworthia* could be attenuated at different level in the gut mucosa.

In summary, this GOS-Lu supplementation experiment created a distinct distribution of main taxonomic families in gut microbiota, in contrast to feed control diet (**Figure 10**).

Based on these results, it can be noted that the addition of the prebiotic GOS-Lu to the animal diet was able to diminish colon tumors to a greater extent (57.5%, **Figure 3**), an effect that can be mainly attributed to an increase in propionate gut concentrations. This SCFA shows antitumor properties (induction of apoptosis in colon cancer cells) (Ooi et al., 2010), and its higher concentrations in the GOS-Lu cohort can be associated to increased populations of bacterial lineages described as good propionate producers, such as *Bacteroides, Paraprevotella* (both *Bacteroidetes* genera) and *Phascolarctobacterium* (a *Firmicutes* genus).

Also, the protective role of GOS-Lu supplementation can be associated with a reduction in gut pro-inflammatory bacterial populations, such as *Desulfovibrio* (a *Proteobacteria* genus), but above all, it can be associated with the greatest abundance of bacterial genera with strong anti-inflammatory and antitumor properties, as *Bifidobacterium* (an *Actinobacteria* genus) and *Faecalibacterium* (a *Firmicutes* genus).

## Conclusion

Based on these results, GOS-Lu can be described as a prebiotic mixture with protective effects against CRC onset and progression in this animal model (57.5% reduction in colorectal tumors), which opens the way to its wide use in human populations as a food supplement. Also, GOS-Lu supplementation causes changes in intestinal microbiota, favoring anti-inflammatory taxons and reducing pro-inflammatory genera.

## Author Contributions

AC, FL, and FM conceived and designed the experiments. JF and AO performed the experiments. JF, CV, and FL analyzed the data. FM and AO provided the GOS-Lu prebiotic. AC, JF, FL, FM, AO, and CV wrote the paper.

## Conflict of Interest Statement

The authors declare that the research was conducted in the absence of any commercial or financial relationships that could be construed as a potential conflict of interest.

## Footnotes

1 https://www.envigo.com/resources/data-sheets/2014s-datasheet-0915.pdf

## Supplementary Material

TABLE S1 Phyla, families and genera data for each of the animals used in this study.

TABLE S2 Raw data on animals weight, caecums weight, number of Peyer’s patches, number of polyps, total tumoral area and short fatty acids concentrations for each of the animals used in this study.
